# Stimulating Effect of *Trichococcus flocculiformis* on a Coculture of *Syntrophomonas wolfei* and *Methanospirillum hungatei*

**DOI:** 10.1128/aem.00391-22

**Published:** 2022-06-14

**Authors:** Anna Doloman, Sjef Boeren, Charles D. Miller, Diana Z. Sousa

**Affiliations:** a Laboratory of Microbiology, Wageningen University & Research, Wageningen, The Netherlands; b Laboratory of Biochemistry, Wageningen University & Research, Wageningen, The Netherlands; c Department of Biological Engineering, Utah State University, Logan, Utah, USA; d Centre for Living Technologies, Alliance TU/e, WUR, UU, UMC Utrecht, Utrecht, The Netherlands; University of Nebraska—Lincoln

**Keywords:** *Syntrophomonas*, anaerobic digestion, cocultures, methane, methanogens

## Abstract

Syntrophic anaerobic consortia comprised of fatty acid-degrading bacteria and hydrogen/formate-scavenging methanogenic archaea are of central importance for balanced and resilient natural and manufactured ecosystems: anoxic sediments, soils, and wastewater treatment bioreactors. Previously published studies investigated interaction between the syntrophic bi-cultures, but little information is available on the influence of fermentative bacteria on syntrophic fatty acid oxidation, even though fermentative organisms are always present together with syntrophic partners in the above-mentioned ecosystems. Here, we present experimental observations of stimulated butyrate oxidation and methane generation by a coculture of Syntrophomonas wolfei with any of the following methanogens: Methanospirillum hungatei, Methanobrevibacter arboriphilus, or Methanobacterium formicicum due to the addition of a fermentative Trichococcus flocculiformis strain ES5. The addition of *T. flocculiformis* ES5 to the syntrophic cocultures led to an increase in the rates of butyrate consumption (120%) and volumetric methane production (150%). Scanning electron microscopy of the most positively affected coculture (S. wolfei, *M. hungatei*, and *T. flocculiformis* ES5) revealed a tendency of *T. flocculiformis* ES5 to aggregate with the syntrophic partners. Analysis of coculture’s proteome with or without addition of the fermentative bacterium points to a potential link with signal transducing systems of *M. hungatei*, as well as activation of additional butyryl coenzyme A dehydrogenase and an electron transfer flavoprotein in S. wolfei.

**IMPORTANCE** Results from the present study open doors to fascinating research on complex microbial cultures in anaerobic environments (of biotechnological and ecological relevance). Such studies of defined mixed populations are critical to understanding the highly intertwined natural and engineered microbial systems and to developing more reliable and trustable metabolic models. By investigating the existing cultured microbial consortia, like the ones described here, we can acquire knowledge on microbial interactions that go beyond “who feeds whom” relations but yet benefit the parties involved. Transfer of signaling compounds and stimulation of gene expression are examples of indirect influence that members of mixed communities can exert on each other. Understanding such microbial relationships will enable development of new sustainable biotechnologies with mixed microbial cocultures and contribute to the general understanding of the complex natural microbial interactions.

## INTRODUCTION

**FIG 1:**
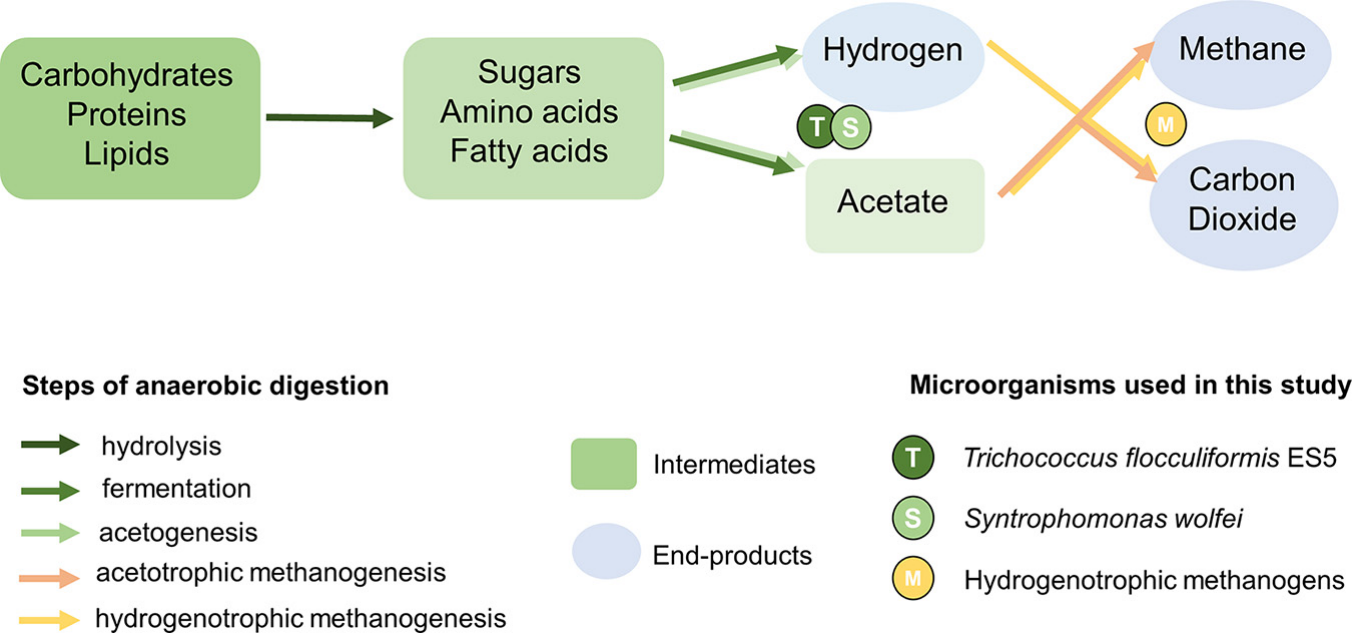
Steps of anaerobic digestion with positions of the microorganisms used in this study.

Complex organic matter in anaerobic natural and engineered environments is effectively transformed into a mixture of carbon dioxide and methane gases (biogas) in a biological process called anaerobic digestion (AD). This process has great importance for global carbon cycling and ensures carbon capture and recovery from waste organic material (such as industrial, municipal, and agricultural waste and wastewater). Since AD is a biological process, it is dependent on smooth cooperation between multiple microorganisms, each carrying out specific biochemical conversions in a chain of AD reactions (Fig. 1). Initial decomposition and hydrolysis of complex organic matter in AD is often accomplished by substrate-specific species of fermentative bacteria that do not rely on other players of the AD chain. On the other hand, subsequent conversion of the organic acids from complex matter often requires an assemblage of microorganisms that heavily depend on each other for survival. These microorganisms are often found to be in syntrophic relationships with terminal players of the AD chain, methanogenic archaea (1). By consuming bacterially produced hydrogen, hydrogenotrophic archaea make the reaction of oxidizing fatty acids thermodynamically feasible, allowing syntrophic bacteria to gain energy for the cellular growth and continue conversion of organic acids (2). Failure to sustain such syntrophic cooperation of microorganisms by inhibiting methanogenic archaea often leads to a break in the AD conversion chain: accumulation of volatile fatty acids, a decrease in pH, and cessation of methane production (3). Industrial biological waste and wastewater treating installations are especially prone to such failures and suffer economically from process instability.

While many studies have investigated syntrophic bacterial-archaeal interplay (4, 5), little to no attention has been paid to the potential influence of fermentative bacteria “up the AD chain” on the stability and efficiency of organic acid digesting partnerships. Fermentative bacteria in the AD chain remain exclusively associated with the upstream provision of the oxidized organic compounds (amino and fatty acids) as growth substrates and energy donors to the microorganisms downstream in the process. Potential influence of fermentative bacteria secondary metabolites (exopolymeric substances, quorum-sensing molecules, etc.) on the syntrophic associations downstream remains unstudied, even though such influence is plausible to occur, especially in complex biofilm associations (6, 7). Such a gap in the studies of AD inevitably leads to truncated knowledge of the entire process, adversely affecting the end goal of making AD stable in industrial setups to ensure a consistent supply of the renewable energy source in the form of biogas. Investigating and understanding a potential multispecies interplay in AD can help us to better understand the functionality of the complex microbial communities, where exchange of metabolites goes beyond thermodynamic benefit.

To investigate a potential effect of adding fermentative microorganisms to syntrophic fatty acid oxidizing cocultures, we chose a bacterium with a versatile substrate range in AD and able to degrade polysaccharides, sugars, and alcohols: a member of the *Trichococcus* genus. *Trichococcus* species have been found in AD bioreactors treating diverse types of wastewaters from, e.g., paper mill (8), sugar refinery (9), and dairy (10) sources. These bacteria were also found in bioreactors supplied with propionate, a fatty acid that is commonly associated with syntrophic methane-producing consortia (11). The pleomorphic nature of cells in this genus and the ability to form long filaments demonstrates the presence of genetic environment-sensing machinery that might include cell-to-cell communication and the secretion of secondary metabolites. Here, we examine the effect of co-cultivating *T. flocculiformis* ES5 with three syntrophic butyrate-oxidizing consortia of Syntrophomonas wolfei and the hydrogenotrophic methanogenic archaea Methanospirillum hungatei, Methanobrevibacter arboriphilus, and Methanobacterium formicicum. Glycerol was supplemented at a very low concentration (1 mM) as a carbon and energy source for *T. flocculiformis* ES5 to investigate the non-substrate-related influence of fermentative bacteria on the syntrophic association. Glycerol is produced during an anaerobic decomposition of phospholipids and triglycerides and is commonly found in a variety of wastewaters (municipal, petrochemical, and pharmaceutical industries, washouts from biodiesel production, etc.) (12–14). The main products of glycerol fermentation by *T. flocculiformis* ES5 are 1,3-propanediol and acetate, although a small amount of formate and lactate can be also observed (8). By growing the syntrophic cocultures together with a glycerol-degrading *Trichococcus* sp., we aimed to compare metabolic activity of the syntrophic cocultures with or without a fermentative partner in the same glycerol- and butyrate-enriched media. The tri-culture that showed the most prominent change in the metabolic rates compared to the bi-culture was additionally subjected to scanning electron microscopy (SEM) and proteome analysis in order to further understand the nature of the *Trichococcus* influence.

## RESULTS

### Growth and morphology of cocultures with or without the addition of *Trichococcus flocculiformis* ES5.

The addition of a fermentative bacterium, *T. flocculiformis* ES5, to all the syntrophic butyrate oxidizing consortia of S. wolfei and hydrogenotrophic methanogenic archaea had a stimulating effect on both butyrate consumption and methane production rates. Figure 1 places the experimentally evaluated triculture consortia into the context of the anaerobic digestion chain. While Syntrophomonas wolfei performs a well-studied oxidation of butyrate (1 mM butyrate → 2 mM acetate) and methanogens autotrophically convert hydrogen into methane, *T. flocculiformis* ES5 ferments glycerol into small quantities of acetate and formate, in addition to the main fermentation product 1,3-propanediol (1 mM glycerol → 0.5 ± 0.1 mM 1,3-propanediol + 0.3 ± 0.2 mM acetate + 0.2 ± 0.1 mM formate) (8).

**FIG 2:**
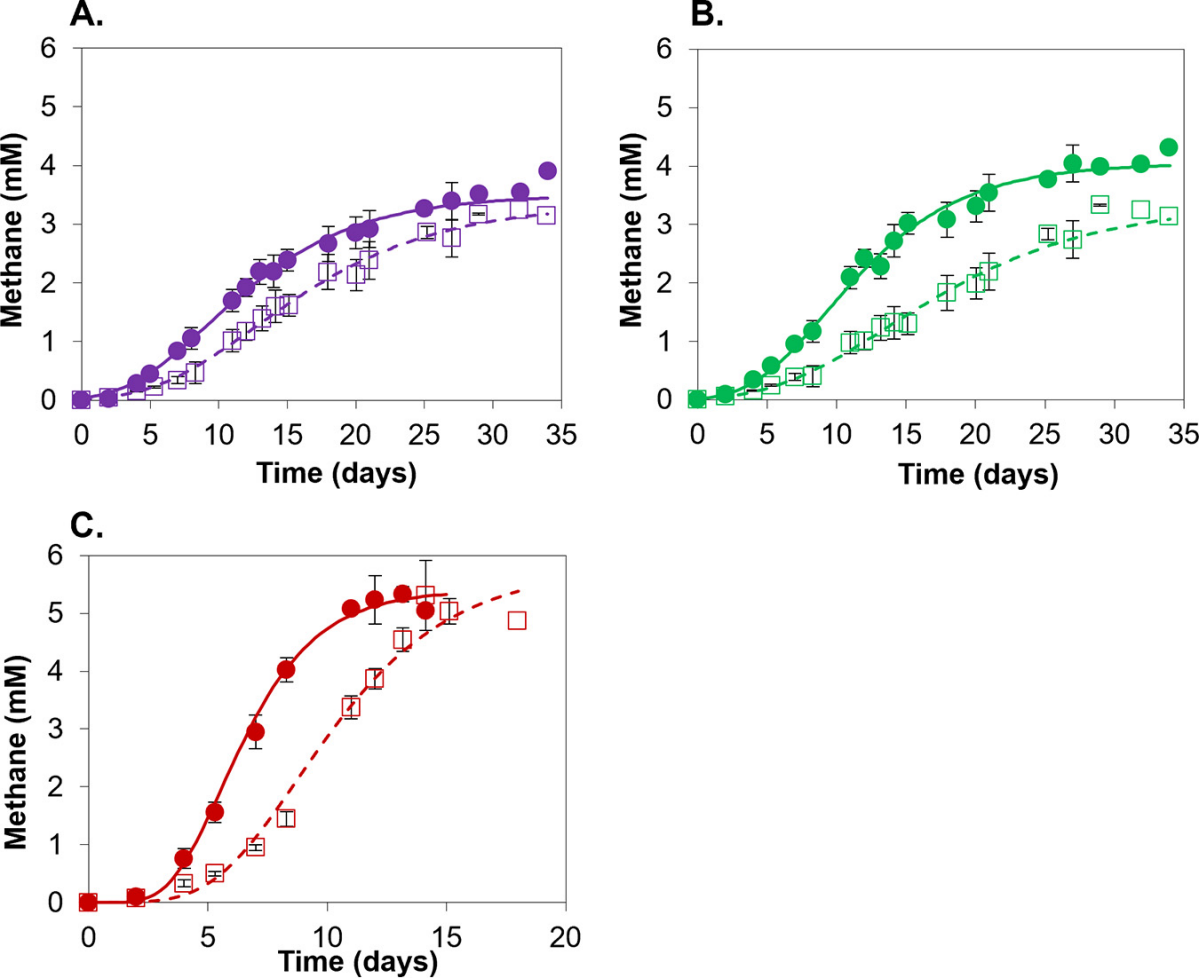
Methane production in cocultures of *T. flocculiformis* ES5, S. wolfei and three different methanogens: *M. arboriphilus* (A), *M. formicicum* (B), and *M. hungatei* (C). Methane production is plotted for tri-cultures with *T. flocculiformis* ES5 (solid line, ●) and bi-cultures without *T. flocculiformis* ES5 (dashed *M. arboriphilus*, *M. formicicum* [B], *M. hungatei* line [C], □). Error bars represent standard deviations between triplicates.

Figure 2 illustrates the differences in the methane productivities of the tested bi- and tricultures, highlighting the stimulating effect of *T. flocculiformis* ES5 on the bi-cultures of S. wolfei with *M. formicicum* and *M. hungatei*. Cocultures with *M. arboriphilus* were also stimulated, but to a much lesser extent. A similar positive stimulatory effect from the addition of *T. flocculiformis* ES5 was also observed for the consumption of butyrate and production of acetate in all the tested combinations of bi- and tri-cultures (see Fig. S1 in the supplemental material). It is important to note that since *T. flocculiformis* ES5 is a fast-growing bacterium, we only used 1 mM glycerol as a substrate for its growth to (i) simulate a plausible scenario in the AD environment, where fermentative growth is much faster than syntrophic, leading to low concentrations of substrate to be fermented, and (ii) to prevent *T. flocculiformis* ES5 (t_d_ = 2.2 h) (8) from overgrowing the population of slow-growing *S. wolfei* (t_d_ = 90 h) (15).

By analyzing the growth kinetics of the bi-cultures (Table 1), we observed that addition of *T. flocculiformis* ES5 to either of the syntroph-methanogen cocultures led to a 60 to 70% decrease in the lag time before methane production occurred, while keeping the maximal concentration of methane unchanged. Volumetric methane production rate was also improved in all tri-cultures compared to the cocultures (130 to 180%) (Table 1; percent changes were calculated by dividing the tri-culture data from the second or third column (γ or *V_m_*) by the corresponding bi-culture data and multiplied by 100%). Calculations of the 95% confidence limits for the predicted kinetic parameters in the bi- and tricultures point to a statistically significant change in the lag phase of the S. wolfei and *M. hungatei* when cultured with *T. flocculiformis* ES5. Notably, the tri-culture with *M. formicicum* had a significantly higher (~2-fold increase) methane production rate compared to the bi-culture pair.

**TABLE 1:** Kinetic parameters from modified Gompertz equation, fit into the methane production data from co-culturing experiments

Coculture	Mean (SE)^*a*^		
	Lag phase (γ) in days	Methane production rate (*V_m_*) in mmol/L/day	Maximal methane concentration (*A*) in mmol/L
S. wolfei + *M. arboriphilus*	5.19 (0.47)	0.168 (0.0095)	3.36 (0.14)
*T. flocculiformis* ES5 + S. wolfei + *M. arboriphilus*	3.04 (0.48)	0.207 (0.0126)	3.61 (0.11)
S. wolfei + *M. formicicum*	5.38 (0.43)	0.15 (0.006)*	3.92 (0.23)
*T. flocculiformis* ES5 + S. wolfei + *M. formicicum*	3.3 (0.55)	0.26 (0.02)*	4.1 (0.14)
S. wolfei + *M. hungatei*	5.96 (0.35)*	0.72 (0.06)	5.5 (0.22)
*T. flocculiformis* ES5 + S. wolfei + *M. hungatei*	3.48 (0.17)*	0.89 (0.05)	5.44 (0.1)

a Asterisks (*) denote statistically significant different means and variances between cocultures with or without *T. flocculiformis* ES5, calculated with a confidence *P* value of <0.05. Numbers in parentheses represent standard errors for the triplicates of the calculated parameters.

Since all the three tested methanogens require small amounts of acetate for cell synthesis and since *T. flocculiformis* ES5 produces acetate from glycerol (Fig. 1), albeit in small amounts, we looked closer into the fate of acetate in the cocultures. Acetate concentrations were significantly higher in the tri-cultures with either of the three methanogens compared to the acetate concentrations under bi-cultures conditions without *T. flocculiformis* ES5 (see Fig. S2). Because acetate is used as a carbon source for all three methanogens and because its greater presence in the tri-cultures might be stimulating methane production, we ran the control experiments with (1.1 mM acetate) or without acetate in the biculture growth media (see Fig. S2). A bi-culture of the syntroph with *M. formicicum* was indeed positively affected (increased methane production) by a slightly elevated starting amount of acetate in the growth media. In contrast, bi-cultures with *M. hungatei* or *M. arboriphilus* were slightly inhibited for methane production due to the addition of acetate. The same effect was observed for the butyrate consumption dynamics (see Fig. S3). We then decided to check the effect of *T. flocculiformis* ES5 specifically on the methanogens (see Fig. S4). The growth medium in this case contained 1.5 mM glycerol and had a headspace of H_2_/CO_2_ (80/20 [vol/vol]) at 1.5 bar. However, no significant difference was observed in the methane generation from either of the *T. flocculiformis* ES5/methanogen combinations. An additional control tested was a bi-culture of *T. flocculiformis* ES5 with S. wolfei (using the same medium formulation as in all of the syntrophic-methanogenic experiments, with or without ES5 cocultures). However, as expected, S. wolfei did not grow, since *T. flocculiformis* ES5 cannot consume hydrogen/formate released from butyrate oxidation (no changes were detected in the initially produced 1.1% [vol/vol] hydrogen and 0.5 mM formate in these cocultures due throughout the 2 weeks of incubation).

Small quantities of formate were detected in the tri-cultures of syntrophic partners with *T. flocculiformis* ES5 (see Table S2) during the first 10 days of microbial growth. The amounts of formate were greater for the tri-culture with *M. hungatei* by 20 to 50% compared to the quantities in cocultures with the other two methanogens. Cocultures with *M. formicicum* had the smallest amounts of formate. In contrast to the tri-cultures, no formate was detected in the bi-cultures without *T. flocculiformis* ES5.

**FIG 3:**
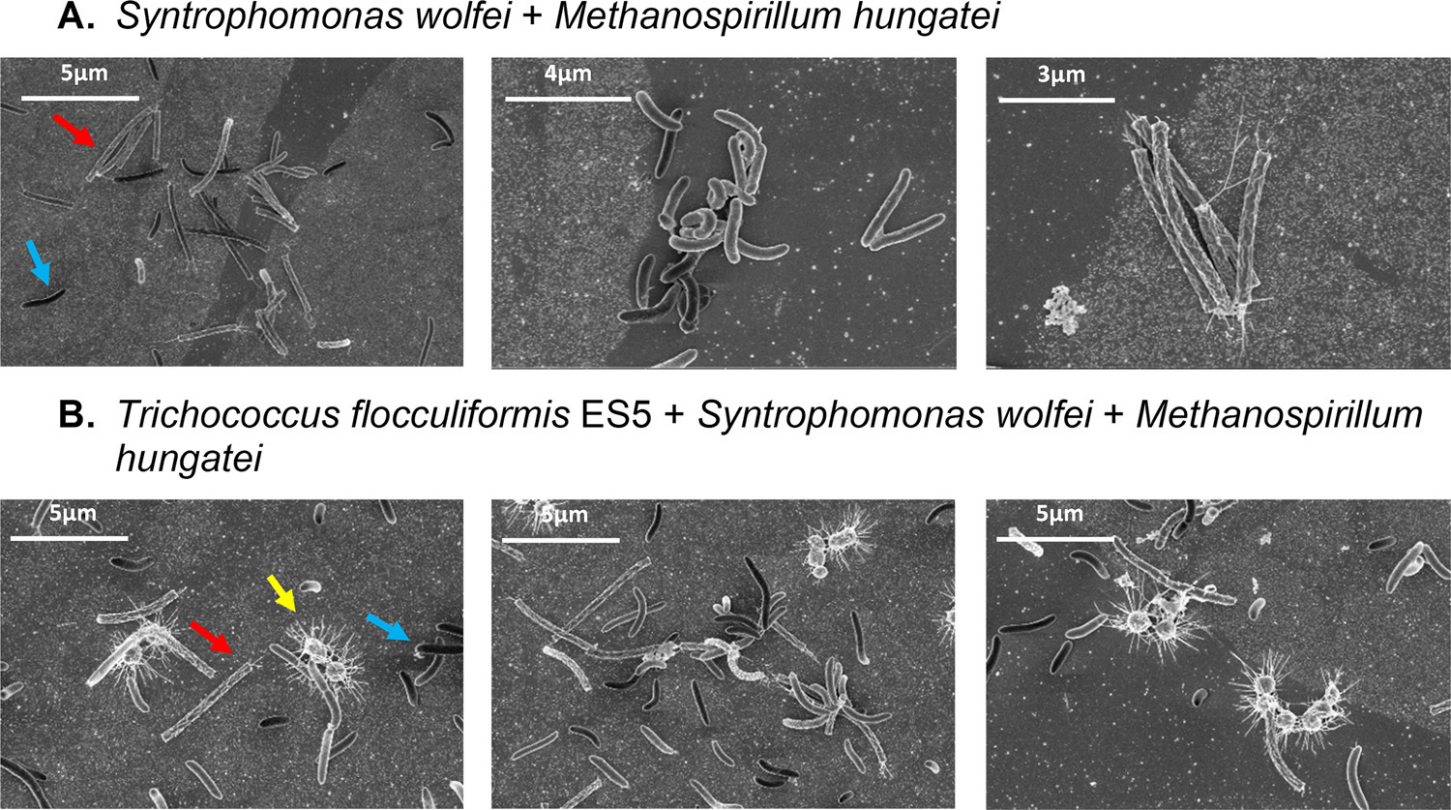
SEM of cocultures of S. wolfei and *M. hungatei* (A) and tri-cultures of *T. flocculiformis* ES5, S. wolfei, and *M. hungatei* (B). Colored arrows point to the three studied microorganisms with distinct morphologies: red for straight long rods of *M. hungatei*, blue for curved rods of S. wolfei, and yellow for cocci of *T. flocculiformis* ES5.

Cocultures with *M. hungatei* were chosen for further close-up investigation of the effect of adding *T. flocculiformis* ES5 to the bi-culture, since they were twice faster in reaching a stationary growth phase compared to cocultures with two other methanogens. Close-up observation of bi-culture and tri-culture morphologies using SEM (Fig. 3) revealed a striking difference in the number of interspecies aggregates between bi-cultures and tri-cultures with the latter having more intraspecies aggregates (Fig. 3B). Notable was the appearance of thin fimbria-like structures surrounding *T. flocculiformis* ES5 cells and their connection with cells of either S. wolfei or *M. hungatei*. A similar *Trichococcus* cell morphology was reported for other species (11, 16), and this might be a layer of exopolymeric substances surrounding the cells.

### Proteome analysis of cocultures with or without the addition of *Trichococcus flocculiformis* ES5.

To further explore the causes of the changed methane production in the syntrophic cocultures due to the addition of *T. flocculiformis* ES5, we performed differential proteomics to compare protein expression profiles of the Syntrophomonas wolfei and Methanospirillum hungatei with or without the addition of the fermentative microorganism. Proteins were extracted from bi- and tri-cultures at the mid-late-exponential methane generation phase (~60% of butyrate was consumed). Using the normalized intensity from the label-free quantitation of the identified proteins, differentially present peptides in two coculture conditions were identified for *M. hungatei* and S. wolfei. In total, 761 proteins were identified under both growth conditions (with or without *T. flocculiformis* ES5) for S. wolfei and 1,370 proteins for *M. hungatei*.

**FIG 4:**
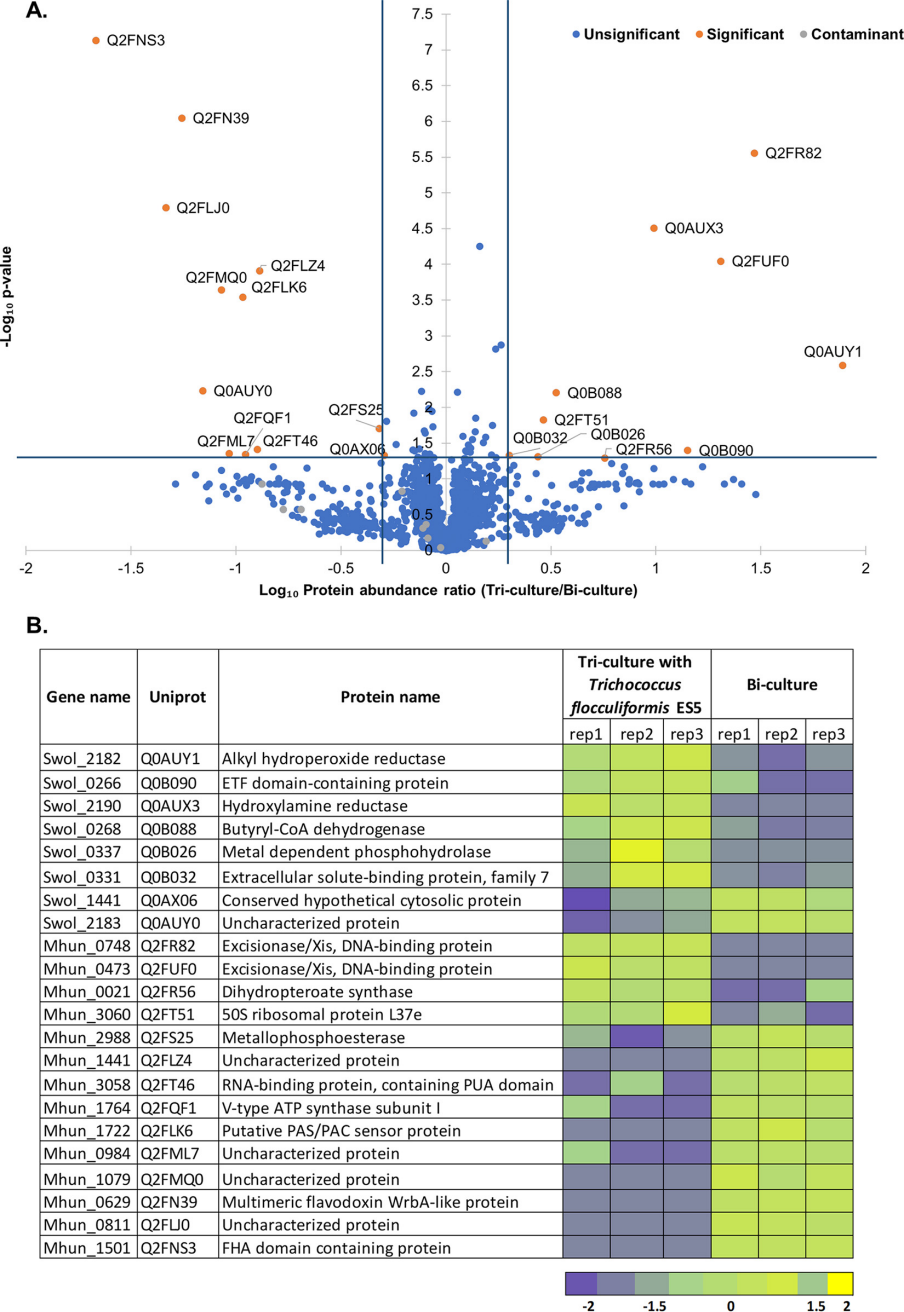
Comparative proteomics results. (A) Volcano plot with comparison of tri- and bi-cultures (with or without *T. flocculiformis* ES5). Blue lines define the limits for the statistical significance in the difference of the detected protein quantities between the two conditions. (B) Identification of the predicted function of significantly different proteins depicted in the volcano plot. Protein abundance levels are shown after Z-score normalization. The color intensity indicates the degree of protein presence, where high relative abundance is indicated in yellow and low relative abundance in blue.

Assuming a 2-fold change in the protein abundance, together with a *P* value below 0.05 as a threshold for significance, 12 proteins were more abundant in bi-cultures of *M. hungatei* and S. wolfei than in tri-cultures, while 10 proteins were more abundant in tri-cultures of syntrophs with *T. flocculiformis* ES5 than in bi-cultures (Fig. 4).

### (i) Differentially abundant proteins of *M. hungatei*.

Of the differentially present proteins belonging to *M. hungatei*, 10 were more abundant in bi-cultures of *M. hungatei* and S. wolfei, while 4 were more abundant in tri-cultures of syntrophs with *T. flocculiformis* ES5. Five of the differentially abundant proteins were poorly characterized or had an unknown function, according to a UniProt/InterPro scan: proteins Mhun_1441, Mhun_1079, and Mhun_0984. The rest of the differentially present proteins belonging to the *M. hungatei* and present in higher quantities under bi-culture conditions were as follows: Mhun_0629 (multimeric flavodoxin WrbA-like protein), Mhun_2988 (metallophosphoesterase with a conserved CHAD domain associated with chelating of metals), Mhun_3058 (RNA-binding protein, containing PUA domain), Mhun_0811 (no identified function or domain), Mhun_1501 (FHA-domain containing protein, which can act as a transcription factor for flagellum-like proteins), and Mhun_1722 that is a signal-sensing PAS/PAC domain, located 58 bp upstream of an operon coding for signal transduction histidine kinase and response regulator receiver domain protein (CheY-like).

Protein Mhun_1764 (V-type ATP synthase subunit I) marginally passed the significance criteria for differential abundance under bi-culture conditions. It is annotated as an integral component of a membrane, performing proton-transporting function, and is in the same operon with H^+^-transporting two-sector ATPase (Mhun_1763).

Overall, it seems that the majority of the *M. hungatei* proteins that were more abundant under bi-culture growth conditions are part of signal transducing or receiving systems.

A small number of differentially abundant proteins of *M. hungatei* under the tri-culture conditions were two excisionase/Xis, DNA-binding proteins of the repair system (Mhun_0748 and Mhun_0473), a 50S ribosomal protein L37e (Mhun_3060), and dihydropteroate synthase (Mhun_0021) involved in the biosynthesis of folate.

### (ii) Differentially abundant proteins of *S. wolfei*.

In contrast to *M. hungatei* having more differentially abundant proteins under bi-culture conditions, S. wolfei had a proteome profile with more changes under the tri-culture conditions with *T. flocculiformis* ES5.

Three proteins associated with substrate (butyrate) consumption and uptake were found to be abundant under tri-culture conditions: substrate binding protein of a TRAP-type transport system (Swol_0331), electron transfer flavoprotein (Swol_0266) and butyryl coenzyme A (butyryl-CoA) dehydrogenase (Swol_0268) (Fig. 4A; see also Table S1). Proteins Swol_0266 and Swol_0268 are in the same operon, while their isoforms, produced from genes located at a different chromosomal location, were experimentally validated to be critical for the syntrophic growth butyrate conversion step (17, 18). Butyryl-CoA dehydrogenase (Swol_0268) catalyzes conversion of butyryl-CoA to crotonyl-CoA and passes the electrons to electron-transfer flavoproteins (Swol_0266). The originally characterized butyryl-CoA dehydrogenase (Swol_2052) by Schmidt et al. (17) was also detected in the protein extract from our mid-exponential-phase grown cocultures, and its level of abundance was similar for both tri-culture and bi-culture conditions, although at a higher level, compared to the Swol_0266 and Swol_0268 complex (log_10_ [protein LFQ] of 9 VS 11) (see Table S1). As can be seen from Table S1, all the proteins and their isoforms involved in the β-oxidation of butyrate to acetate are highly abundant under both coculture conditions. The higher abundance of Swol_0266 and Swol_0268 under tri-culture conditions might hint at the activation of an additional functionally redundant butyrate-oxidizing activity in the S. wolfei due to the presence of *T. flocculiformis* ES5 in the coculture. The presence of a substrate-binding protein of a TRAP-type transport system (Swol_0331) in higher quantities under tri-culture conditions may explain the observed faster butyrate consumption in tri-cultures (see Fig. S1).

The other three proteins that were more abundant under tri-culture conditions do not seem to have a substrate consumption-related activity: hydroxylamine reductase (Swol_2190), metal-dependent phosphohydrolase (Swol_0337), and alkyl hydroperoxide reductase (Swol_2182). The search for the conserved domains with known functions in an InterPro scan and using Pfam did not give any clear function-related clues: Swol_0337 might, for instance, be involved in either a protein-binding or a signal-transducing function. Alkyl hydroperoxide reductase (Swol_2182) can be involved in mediating defense against oxidative damage or help to maintain cell redox homeostasis. However, it is not clear why it would be present in higher quantities in a tri-culture with *T. flocculiformis* ES5.

Under bi-culture conditions, only two proteins were identified as more abundant for S. wolfei: Swol_1441 has similarities with homocysteine biosynthesis enzyme, involved in sulfur-incorporation, while Swol_2183 is uncharacterized and has a signal peptide region (23 amino acids at the N terminus). Swol_2183 also has two copies of an S-layer homology domains, with a 83-amino-acid fragment that contains another distinct surface protein associated domain (Big_2 family) tentatively playing role in bacterial cell adhesion. Thus, Swol_2183 might be a cell-wall-associated protein of unknown signaling and/or adhesive function that is more relevant for the bi-culture growing condition with *M. hungatei* than for a tri-culture with *T. flocculiformis* ES5 and *M. hungatei*.

## DISCUSSION

In this study, we successfully demonstrated that AD systems can have microbial interconnections that go beyond thermodynamic benefit of the obligatory syntrophic fatty acid oxidation (the well-studied associations of fatty acid-oxidizing bacteria and methanogens). We observed a positive influence that seemingly metabolically unrelated fermentative bacterium, *T. flocculiformis* ES5, can have on the syntrophic cocultures of S. wolfei and the hydrogenotrophic methanogenic archaea *M. hungatei*, *M. arboriphilus*, and *M. formicicum*. All three butyrate-oxidizing syntrophic partnerships were stimulated by the presence of *T. flocculiformis* ES5: tri-cultures had higher CH_4_ production rates and decreased lag times of butyrate oxidation compared to the bi-cultures.

Closeup investigation of the fastest growing coculture of S. wolfei with *M. hungatei* and *T. flocculiformis* ES5 revealed differentially abundant proteins that are specific to tri-culture conditions. Among these were proteins involved in substrate utilization by S. wolfei. We identified a high abundance of five isoforms of butyryl-CoA dehydrogenase that catalyze the second step of butyrate oxidation (conversion of butyryl-CoA to crotonyl-CoA) (see Table S1). While four of them were highly abundant under both conditions, the fifth, Swol_0268, was specifically highly abundant under tri-culture conditions. The overabundance of this protein can possibly explain the shorter lag phase in butyrate consumption of tri-cultures. However, it is not clear whether the presence of *T. flocculiformis* ES5 alone was activating production of this enzyme. As can be noted from the Fig. S1, butyrate consumption rates were similar regardless of presence of *T. flocculiformis* ES5 in the syntroph-methanogen coculture (1.7 mM/day). It is the lag time before butyrate consumption that was almost twice shorter in the presence of *T. flocculiformis* ES5 (Table 1). The fact that the concentration of acetate, the main metabolic product of butyrate oxidation, is also significantly increased under triculture conditions (see Fig. S1) may be an indirect indication of a stimulating effect *T. flocculiformis* ES5 exerts on the S. wolfei. The increased amount of acetate cannot be attributed to *T. flocculiformis* ES5 itself, since stoichiometrically, the bacterium only produces 0.3 mM acetate per 1 mM glycerol, while S. wolfei produces 2 mM acetate per 1 mM butyrate (Fig. 1). Thus, the stimulative effect of *T. flocculiformis* ES5 on *S. wolfei* is more realistic due to the observed increased abundance of the electron transfer flavoprotein (Swol_0266) and butyryl-CoA dehydrogenase (Swol_0268) under tri-culture conditions, in addition to the other butyryl-CoA dehydrogenases under bi- or triculture conditions (Swol_2052, Swol_0788, Swol_0488, and Swol_1841) (see Table S1).

Increased butyrate oxidation by S. wolfei might be also partially influenced by the more active hydrogen scrubbing by the methanogens, which in turn might be also stimulated by the *T. flocculiformis* ES5. When grown on glycerol, *T. flocculiformis* ES5 produces small quantities of acetate (~0.2 mM/mM glycerol) and formate (~0.1 mM/mM glycerol) (8). While small quantities of acetate are needed for hydrogenotrophic methanogens to synthesize cell material, formate can be an alternative to H_2_ as electron carrier. As can be seen from the data presented in Table S2, small quantities of formate (up to 2.6 mM) were detected in tri-cultures but not in bi-cultures. Since all the tested methanogens are able to use formate to some extent, the presence of formate in the tri-cultures might explain part of the increased methane production under this condition compared to the methane production rates in bi-cultures (Table 1 and Fig. 2). However, just the addition of *T. flocculiformis* ES5 to the pure cultures of methanogens did not stimulate the additional methane generation as in the tricultures (see Fig. S4), and no formate was detected in the supernatants of *T. flocculiformis* ES5 and methanogen bicultures. Thus, the phenomenon is clearly characteristic specifically to tri-cultures with S. wolfei and *T. flocculiformis* ES5. Small quantities of acetate and formate might have been used to jump start cell growth of methanogens during the early phases of the co-cultivation when hydrogen has not been produced by the S. wolfei yet (the first 24 h of incubation). That might have led to a cascade effect: higher cell counts of methanogens in the first 24 to 48 h resulted in a faster relief of hydrogen partial pressures as soon as S. wolfei started consuming butyrate, consequently leading to a shorter butyrate oxidation lag time (Table 1 and Fig. 2). A plausible follow-up study might involve testing the bi- and tri-cultures of S. wolfei and *T. flocculiformis* ES5 with a methanogenic partner that is unable to consume formate. If indeed formate was responsible for the elevated methane production in the tri-cultures, cocultures with a methanogen deficient in formate dehydrogenase activity will not respond to the presence of *T. flocculiformis* ES5 in a way similar to the methanogens tested in this study. In addition, collecting and analyzing transcriptomes from bi- and tri-culture conditions might be more useful than proteomic studies, since changes in the transcriptome are more sensitive than changes in the proteome to the differential expression of formate dehydrogenase and other membrane-bound proteins.

The miniscule amounts of *T. flocculiformis-*produced formate and acetate might also explain why the protein profile of *M. hungatei* did not change much in the two co-cultivation conditions (Fig. 4). Moreover, the proteins that were affected by the presence of *T. flocculiformis* ES5 were mostly uncharacterized. Closer re-annotation revealed conserved domains in some of those proteins and hints to their potential role in a poorly studied signal transduction of *M. hungatei*. For example, uncharacterized Mhun_1441 belongs to an operon that has only one other gene, Mhun_1440, a “putative PAS/PAC sensor protein.” Interestingly, upstream of this operon is located another operon that also consists of “putative PAS/PAC sensor protein” and “response regulator receiver domain protein (CheY-like).” The protein of interest for the tri-culture condition, Mhun_1441 can be a part of the operon that is part of a two-component signal transduction system, where putative PAS/PAC sensor protein can be involved in small molecule interaction. Another potential signal transduction-related protein, which was abundant in bi-culture conditions, was Mhun_0984: uncharacterized protein which is conserved among a couple of methanogenic genomes belonging to a *Methanospirillum* spp. and *Methanomicrobiales* spp. InterPro scan revealed the presence of a potential signal peptide at the first 36 amino acids, with the rest of the protein located in the non-cytoplasmic region. The gene encoding Mhun_0984 has two annotated genes upstream; 49 bp apart is a gene encoding DNA replication helicase protein MCM, and 60 bp upstream that gene is a two-component transcriptional response regulator from the LuxR family. Further in the same cluster are located genes encoding signal transduction system and chemotaxis response regulator cheB. Even though protein Mhun_0984 is not in the operon with either of the upstream genes, it might be involved in a protein-protein interaction with either of the groups.

The fact that *M. hungatei* signal transduction system might be stimulated or influenced by the presence of *T. flocculiformis* ES5 is intriguing, since SEM of the tri-cultures showed a very close proximity of *T. flocculiformis* ES5 cells to the cells of *M. hungatei* (as well as to the cells of S. wolfei) (Fig. 3). A recent speculative study on the electroconductivity of *Trichococcus* species (19) suggests that *T. flocculiformis* ES5 cells are enhancing electron transfer in the aggregates of the butyrate-oxidizing partners, although this hypothesis needs to be further tested. Plausible scenarios of what cells of *T. flocculiformis* ES5 are doing when physically close to either syntrophs or methanogens might be that *T. flocculiformis* ES5 is acting as a conductive material between S. wolfei and a methanogenic partner (20) or that *T. flocculiformis* ES5 is somehow stimulating membrane-bound electron-transport proteins. A few previous studies with supplementation of methanogenic communities with conductive materials have demonstrated resulting decreased lag times for methane generation, as well as significantly increased methane production rates (21, 22). For example, mixing granular activated carbon, Geobacter metallireducens, and acetoclastic Methanosarcina barkeri led to methane production in the presence of CO_2_, without acetate (23), assuming hydrogen is being transferred directly between the microbial species. Another study demonstrated a stimulating effect of magnetite additions on the mixed anaerobic sludge consortium, leading to decreased lag times in the oxidation of propionate and increased rates of methane generation (24). All of these studies might provide evidence for direct electron transfer, but no studies of this type were performed with pure cultures of hydrogenotrophic methanogens to specifically study and disseminate the potential stimulating effect.

Chemotaxis of methanogens should also be considered as a plausible explanation for the increased methane generating behavior observed in tri-cultures with *T. flocculiformis* ES5. For example, analysis of the publicly available genome of *M. hungatei* from the NCBI database demonstrates the presence of a full operon assemblage for flagellum (archaellum) synthesis. The structure of archaella from *M. hungatei* has been recently investigated in detail with cryo-EM imaging (25) and has been proven to demonstrate electrical conductivity (26). Archaella resemble bacterial type IV pili and are responsible for the chemotactic movement of the cell in a carbon substrate gradient or toward a growth-limiting electron donor (H_2_) (27). There is a documented chemotactic response of *M. hungatei* toward acetate (28), which is exponentially increased up until 20 mM acetate in media. Since in our growth trials, the acetate concentration was above 20 mM within the first week of coculturing *M. hungatei* and S. wolfei (see Fig. S2) and since the acetate concentration was significantly higher in the tri-cultures with *T. flocculiformis* ES5, chemotaxis toward acetate might indeed be a plausible explanation for the increased methane generation that we observed. However, we did not detect any known chemotaxis-associated proteins to be more abundant in the tri-culture conditions (Fig. 4). As for the other two methanogens that were not investigated in close details by SEM, neither *M. formicicum* nor *M. arboriphilus* have archaellum synthesis operons. *M. arboriphilus* has a single gene responsible for the synthesis of prepilin peptidase, but it might not be enough for any chemotactic response.

**FIG 5:**
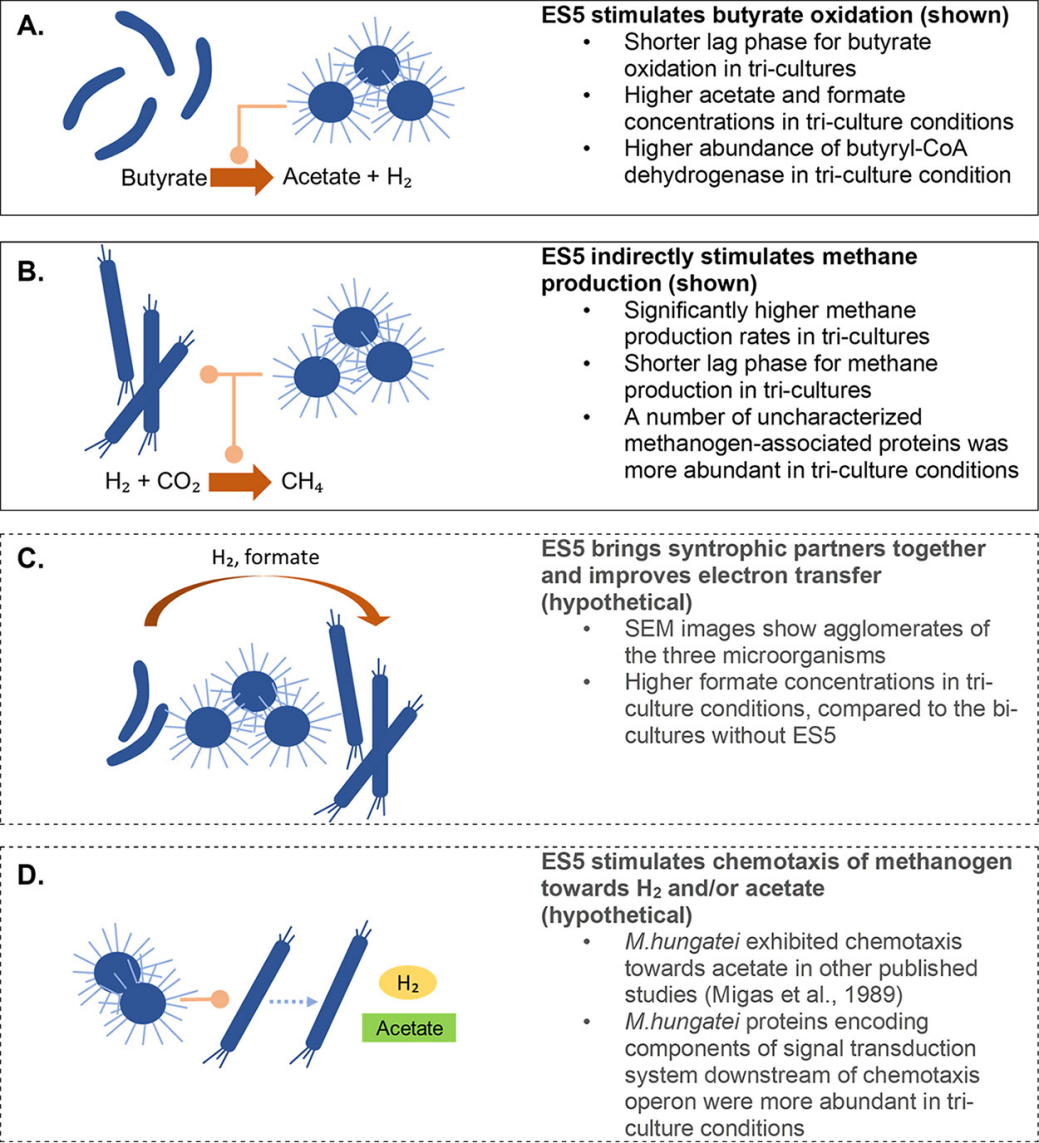
Possible mechanisms for interaction between *T. flocculiformis* ES5 and the syntrophic partners (proved [A and B] and to be tested [C and D]).

A summary of all of the hypothesized relationships that *T. flocculiformis* ES5 might have with syntrophic partners is depicted in Fig. 5. This study opens a door to the fascinating research on complex microbial cultures in AD and beyond. With respect to the proposed explanations of the observed stimulating effect that the fermentative microorganism *T. flocculiformis* ES5 has on obligately syntrophic partnerships, it is becoming increasingly evident that complex microbial systems, natural or engineered, are still largely misunderstood. With the increased use of the modern omics tools and a thirst to assess the microbial potential of every ecological niche on Earth, it is important to look beyond “who feeds whom” scenarios of microbial interactions. As suggested more than a decade ago, the reason we cannot isolate the majority of microorganisms under laboratory conditions is because these potential isolates need to have something more than the right electron donors/acceptors and redox conditions (29): they might be lacking signals from their natural microbial neighbors. Newer studies take steps in these directions and test various microfluidics devices and membrane-diffusion bioreactors to access microorganisms from their natural habitats with the natural stimulators of their growth (30). However, we need studies of the mixed microbial populations, where both physiological and genetic stimuli are closely investigated. Studying more complex and not straightforward microbial relationships will aid future efforts to help us understand the highly intertwined natural microbial systems and to develop more reliable and trustable metabolic models. Understanding complex microbial relationships will enable the development of newer sustainable biotechnologies for a safer environment not only for humans but also for the preservation of natural living diversity.

## MATERIALS AND METHODS

### Growth of pure cultures, bi-cultures, and tri-cultures of syntrophic microorganisms.

Pure cultures of three methanogens were obtained from German Collection of Microorganisms and Cell Culture: Methanospirillum hungatei strain JF1 (DSM 864), Methanobrevibacter arboriphilus strain SA (DSM 7056), and Methanobacterium formicicum strain MF (DSM 1535). A pure culture of Syntrophomonas wolfei strain G311 was kindly provided by Michael J. McInerney, the University of Oklahoma. A Trichococcus flocculiformis ES5 (DSM 23957) culture was originally isolated in our laboratory from granulated methanogenic sludge, treating paper mill wastewater (8), and further characterized by Strepis et al. (31).

Bicarbonate-buffered mineral salt medium (32) was used for all microbial cultures. Boiled and N_2_-flushed medium (50 mL) was dispensed into 120-mL serum vials, sealed with black butyl rubber septa, and crimped with aluminum caps. The vial headspace was flushed with H_2_/CO_2_ (for growing pure cultures of methanogens) or N_2_/CO_2_ (for syntrophic cocultures and pure *T. flocculiformis* ES5 cultures) at 80/20 (vol/vol) and pressurized to 1.5 bar. Medium was autoclaved at 120°C for 20 min. Before inoculation medium was supplemented with filter-sterilized vitamins solution (32) and reduced with Na_2_S×*x*H_2_O (*x* = 9 to 11 mol; added to a final concentration of ~1 mM).

Each of the three syntrophic bi-cultures was constructed by mixing a 10% (vol/vol) inoculum of the pre-grown pure culture of S. wolfei and methanogens. For tri-cultures *T. flocculiformis* was additionally inoculated in 1% (vol/vol). All bi- and tri-cultures were prepared in triplicates. All the experiments with bi- and tri-cultures were supplemented with butyrate (20 mM), glycerol (1 mM), yeast extract (0.05 g/L), and sodium acetate (2 mM). Media for the growth of pure cultures of methanogens were supplemented with sodium acetate (2 mM) only. Pure cultures of Syntrophomonas wolfei G311 and Trichococcus flocculiformis ES5 were pre-grown on the basal medium supplemented with sodium crotonate (20 mM) and glycerol (20 mM), respectively. Additional controls were made for all three types of pure cultures grown on media with butyrate, glycerol, yeast extract, and sodium acetate mixed. Pure cultures of methanogens were incubated on a shaker platform (180 rpm). Cultures of *T. flocculiformis* ES5 and S. wolfei were not shaken during incubation. All cultures were grown at 37°C in the dark.

### Analytical methods.

Gaseous compounds (H_2_, CH_4_) were analyzed by gas chromatography on a CompactGC^4.0^ (Interscience, Breda, Netherlands) equipped with a thermal conductivity detector. Argon gas was used as a carrier gas at a flow rate of 1 mL min^−1^. 0.2 mL of gas sample was injected onto a preseparation Carboxen 1010 column (3 m × 0.32 mm), followed by a separation on a Molsieve 5A column (30 m × 0.32 mm). The temperatures in the injector, column, and detector were 100, 140, and 110°C, respectively. Volatile fatty acids were quantified using a Shimadzu HPLC (Duisburg, Germany), equipped with a Shodex column (SH-1011), and UV/RID detectors. A flow rate of 1 mL min^−1^ was used with sulfuric acid (0.01 N) as the mobile phase and a column temperature set at 45°C.

### Scanning electron microscopy.

Stationary-phase grown cultures were subjected to SEM. Centrifugation of the samples was avoided during sampling to prevent unnatural clumping of bacteria and thus interference with the detection of any aggregates. Instead, aliquots of culture samples were directly mounted on coverslips coated with poly-l-lysine (Corning BioCoat; Corning Life Sciences, Tewksbury, MA) and fixed with 3% (vol/vol) glutaraldehyde and 1% (vol/vol) OsO_4_. Samples were fixed for 1 h at room temperature and then dehydrated in graded ethanol solutions in water (10, 30, 50, 70, 80, 90, 96, and 100%) for 10 min each and critical point dried with liquid carbon dioxide by using an EM CPD300 automated critical point dryer (Leica, Wetzar, Germany). Cells were studied with an FEI Magellan 400 scanning electron microscope.

### Protein extraction and proteomics analysis.

Protein extraction was performed on triplicates of mid- to late-exponential-phase grown co- and tri-cultures. Triplicates of 250-mL cultures grown in 500-mL sealed glass bottles were subjected to protein extraction for each tested condition. Cultures were cooled down to 4°C and harvested by centrifugation (24,471 × *g*, 10 min). After the culture pellet was washed in phosphate buffer (50 mM), the cells were ruptured by sonication (Sonifier B12; Branson Sonic Power Company, Danbury, CT) in lysis buffer containing 100 mM Tris/HCl (pH 7.5) and 4% SDS, with the addition of 1 mM PMSF (phenylmethylsulfonyl fluoride). Extracted protein was quantified with Pierce BCA assay (Thermo Fisher) according to the manufacturer’s protocol. Protein extracts were stored at −80°C until sample preparation was continued. Of each sample, 60 μg of protein was denatured by heating at 95°C for 5 min and loaded onto a 4 to 20% precast protein gel, followed by a short 5-min electrophoresis run. Coomassie blue-stained proteins were reduced in 50 mM ammonium bicarbonate (pH 8.0; ABC buffer) with 15 mM dithiothreitol at 45°C for 1 h, washed and alkylated with 20 mM acrylamide in 100 mM Tris-HCl (pH 8.0) (room temperature in the dark for 0.5 h), and washed again. Protein slices were cut from the gel and cut into cubes of ~1 mm^3^. In-gel tryptic digestion was performed by adding 50 μL of 5 ng/μL trypsin (sequencing grade; Roche, Basel, Switzerland) in ABC buffer, followed by incubation of the gel cubes overnight with gentle shaking at 20°C. The peptide mixture was acidified to a pH of 3 by adding 10% (vol/vol) trifluoroacetic acid in water, filtered, concentrated, and reconstituted into 50 μL of 1-mL/L formic acid in water.

A proteome analysis of syntrophically grown *S. wolfei* and *M. hungatei* in bi-culture or tri-culture (with *T. flocculiformis* ES5) was performed with an EASY nanoLC-Q-Exactive HFX MS (Thermo Electron, San Jose, CA). Next, 1.5-μL portions of peptide samples were loaded directly onto a ReproSil-Pur 120 C_18_-AQ analytical column (0.10 × 250 mm, 1.9-μm beads; prepared in-house) at a constant pressure of 825 bar (flow rate, ~700 nL/min) with, as buffer, 1-mL/L formic acid in water and eluted at a flow rate of 0.5 μL/min with a 50-min linear gradient from 9 to 34% acetonitrile in water with 1-mL/L formic acid. An electrospray potential of 3.5 kV was applied directly to the eluent via a stainless-steel needle fitted into the waste line of the micro-cross that was connected between the nLC and the analytical column. Full-scan positive-mode FTMS spectra were measured between *m/z* 380 and 1,400 on at a resolution of 60,000. MS and MS/MS AGC targets were set to 3.106 and 50,000, respectively, or maximum ion injection times of 50 ms (MS) and 25 ms (MS/MS) were used. HCD fragmented (isolation width, 1.2 *m/z*; 24% normalized collision energy) MS/MS scans of the 25 most abundant 2+ to 5+ charged peaks in the MS scan were recorded in data-dependent mode (threshold 1.2E5, 15-s exclusion duration for the selected *m/z* ± 10 ppm).

Protein identification and relative quantitation was performed using MaxQuant software (v.1.6.3.4) (33) with a built-in Andromeda database search algorithm. The following modifications were included into the protein identification and quantification: oxidation (M), acetyl (protein N-term) and deamidation (NQ). Extracted MS/MS spectra were searched against the UniProt *M. hungatei* and S. wolfei protein sequence database. Databases for *M. hungatei* (UP000001941_2020) and S. wolfei (UP000001968_2020) were downloaded in April 2020. Amino acid sequences of known contaminant proteins (e.g., skin and hair proteins, trypsin, and LysC) were included in the contaminants database. The “label-free quantification,” as well as the “match between runs,” options were enabled. Peptides and proteins with a false discovery rate of <1%, and proteins with at least two identified peptides (at least one should be unique and at least one should be unmodified) were accepted. Results showing a normalized label-free quantitation intensity (LFQ) value of 0 for two triplicates were deleted from the results table. The logarithm with a base of 10 was taken from protein LFQ MS1 intensities, as obtained from MaxQuant. Zero “Log LFQ” values that remained after filtering were replaced by a value of 6.6 (a value slightly lower than the lowest measured value) to make sensible ratio calculations possible.

### Growth data analysis and fitting the model.

To mathematically evaluate the influence of *T. flocculiformis* ES5 on cocultures of S. wolfei with three different methanogens, a modified Gompertz equation (equation 1) (34) was used to calculate kinetic parameters and fit into the obtained data on methane production:

(1) $$f\left(t\right)=Ae^{-e^{\frac{v_{m}e}{A}\left(\gamma -t\right)+1}}$$

where *A* is the maximal concentration of methane reached (mmol/L), *V_m_* is the volumetric production rate (mmol/L/day), and γ is the lag time before production occurs (days).

Data were fit into equation 1 by means of nonlinear regression using the Newton algorithm in the NLIN procedure of SAS (35).

### Data availability.

The mass spectrometry proteomics data have been deposited to the ProteomeXchange Consortium via the PRIDE (36) partner repository with the data set identifier PXD033054.
