# Supplementary material for: Stimulating Effect of Trichococcus flocculiformis on a Coculture of Syntrophomonas wolfei and Methanospirillum hungatei
Source: Appl Environ Microbiol. 2022 Jun 14;88(13):e00391-22. doi: 10.1128/aem.00391-22 (PMC9275234; doi:10.1128/aem.00391-22)
Supplement: Supplemental file 1 — Tables S1 and S2 and Fig. S1 to S4. Download aem.00391-22-s0001.pdf, PDF file, 0.2 MB [file aem.00391-22-s0001.pdf]

Stimulating Effect of *Trichococcus flocculiformis* on a Co-culture of *Syntrophomonas wolfei* and *Methanospirillum hungatei*

Anna Doloman, Sjef Boeren, Charles D Miller, Diana Z Sousa

SUPPLEMENTARY INFORMATION

Table S1. Identified proteins of butyrate oxidation pathway in *Syntrophomonas wolfei*, with abundance in two culturing conditions: tri-culture with *Trichococcus flocculiformis* ES5 or bi-culture without *Trichococcus flocculiformis* ES5. Protein abundance levels are shown after Z-score normalization. The color intensity indicates the degree of protein presence, where high relative abundance is indicated in yellow and low relative abundance in blue. Proteins shown in **bold** are statistically differentially abundant in either of the two conditions.

Gene name	Uniprot	Protein name	Related biochemical conversion step	Tri-culture with *Trichococcus flocculiformis* ES5			Bi-culture		
				rep1	rep2	rep3	rep1	rep2	rep3
Swol_0309	Q0B052	Probable butyrate:acetyl-CoA coenzyme A-transferase	Butyrate						
Swol_1147	Q0AXU7								
Swol_1014	Q0AY76								
Swol_0698	Q0AZ32	EtfAB:quinone oxidoreductase	Butyryl-CoA						
**Swol_0268**	**Q0B088**	Butyryl-CoA dehydrogenase							
Swol_2052	Q0AVA8								
Swol_1841	Q0AVW5								
Swol_0788	Q0AYU5		e^-						
Swol_0488	Q0AZN1								
**Swol_0266**	**Q0B090**	**ETF domain-containing protein**	membrane-associated FeS - containing reductase						
etfA	Q0AZ33	Electron transfer flavoprotein subunit alpha							
etfB	Q0AZ34	Electron transfer flavoprotein subunit beta							
Swol_0650	Q0AZ77	Putative crotonase (Crotonyl-CoA hydratase)	Crotonyl-CoA						
Swol_1171	Q0AXS4	3-hydroxyacyl-CoA dehydrogenase							
Swol_0307	Q0B054								
Swol_2030	Q0AVD0								
Swol_0791	Q0AYU2		3-hydroxybutyryl-CoA						
Swol_1934	Q0AVM3	Acetyl-CoA acetyltransferase (Acetoacetyl- CoA thiolase)							
Swol_2051	Q0AVA9	Acetyl-CoA acetyltransferase / 3-ketoacyl-CoA thiolase	Acetoacetyl-CoA						
Swol_0767	Q0AYW6	Phosphotransacetylase							
ackA	Q0AYW5	Acetate kinase	Acetate / ATP						

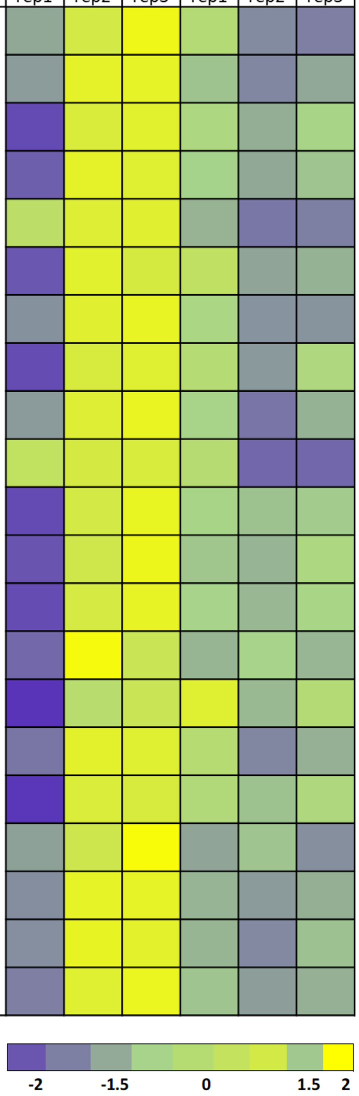

Table S2. Formate in the tri-cultures of *T. flocculiformis* ES5, *S. wolfei* three different methanogens. No formate detected on Day 0 or after Day 10.

Sample\Formate, mM	Day 5		Day 10	
	Average	STDEV	Average	STDEV
ES5 + *S.wolfei* + *M.arboriphilus*	1.1	0.1	1.3	0.1
ES5 + *S.wolfei* + *M.formicicum*	1.3	0	1.6	0.1
ES5 + *S.wolfei* + *M.hungatei*	1.6	0.1	2.5	0.1

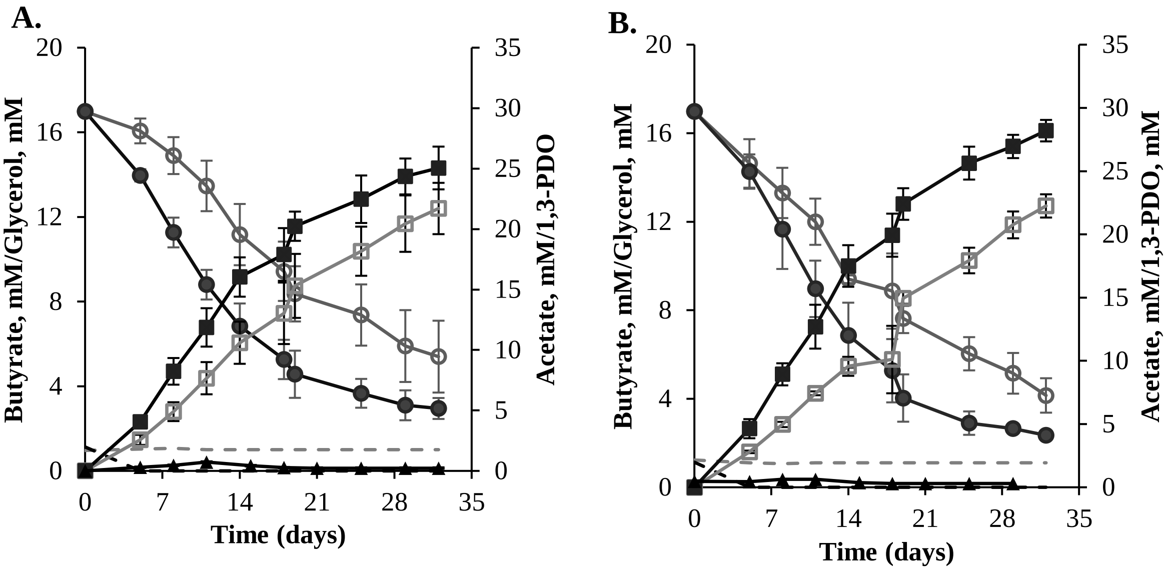

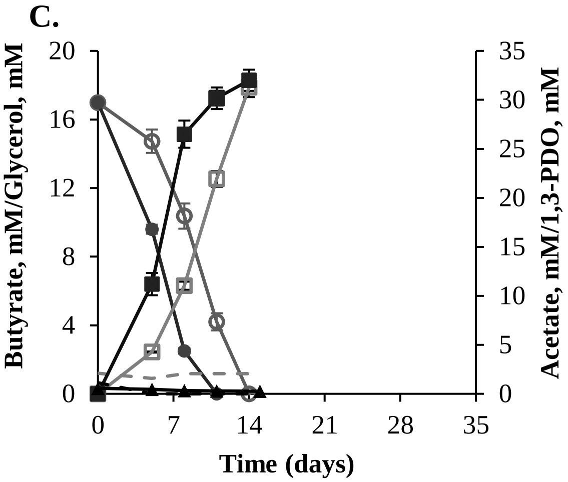

Figure S1. Consumption of butyrate (●, ○), glycerol (-, -) and production of acetate (■, □), 1,3-propanediol (1,3-PDO, ▲) in co-cultures of *S. wolfei* with *M. arboriphilus* (**A**), with *M. formicicum* (**B**), with *M. hungatei* (**C**) and with *T. flocculiformis* ES5 (●, ■, ▲, -) or without *T. flocculiformis* ES5 (○, □, -).

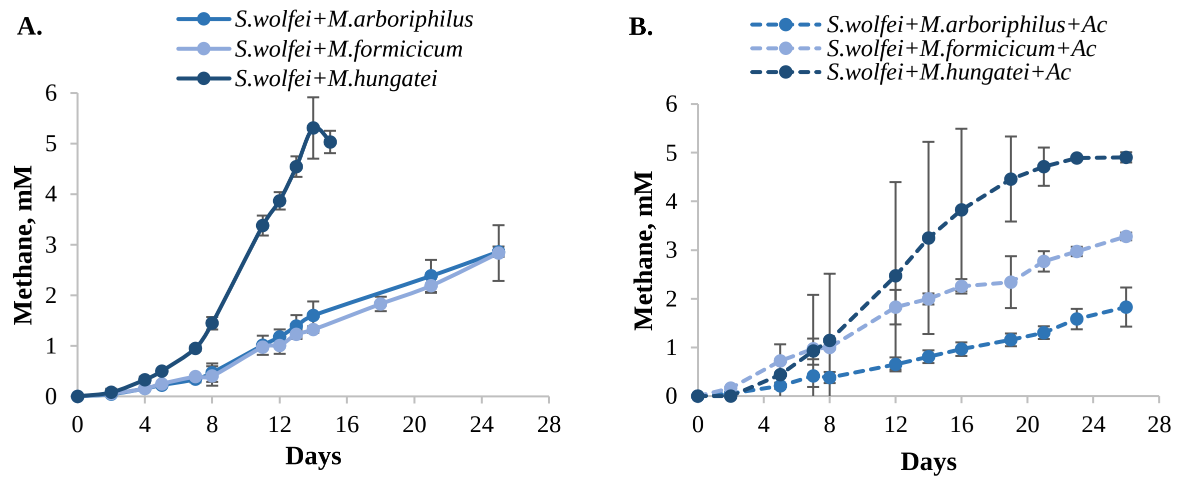

Figure S2. Methane generation in the bi-cultures of *S. wolfei* with *M. hungatei*, *M. arboriphilus*, or *M. formicicum* without (**A**) or with (**B**) 1.1 mM acetate in the starting CP media composition. Error bars represent standard deviations between triplicates (**A**) or duplicates (**B**).

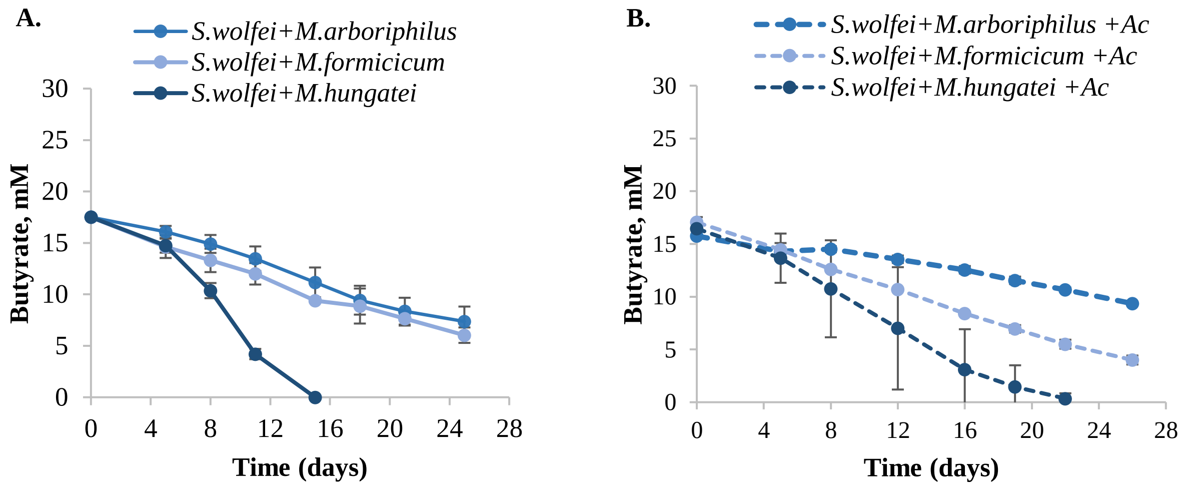

Figure S3. Butyrate consumption in the bi-cultures of *S. wolfei* with *M. hungatei*, *M. arboriphilus*, or *M. formicicum* without (**A**) or with (**B**) 1.1 mM acetate in the starting CP media composition. Error bars represent standard deviations between triplicates (**A**) or duplicates (**B**).

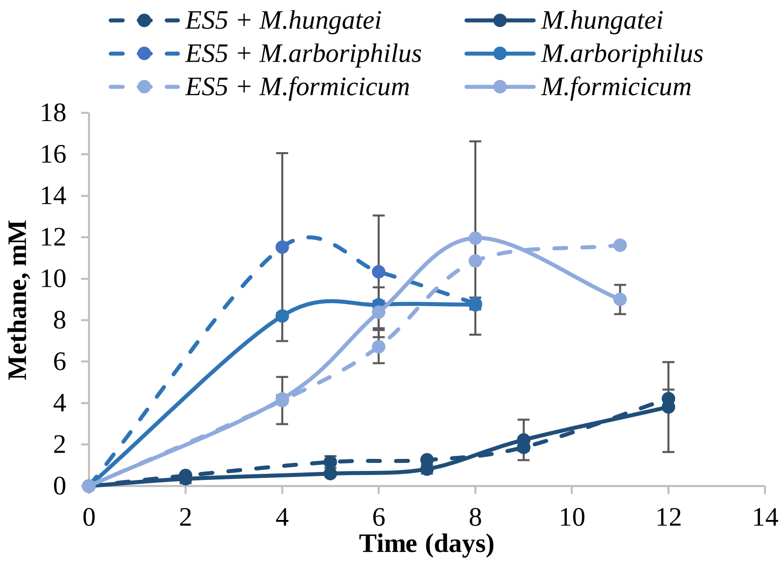

Figure S4. Influence of the *T. flocculiformis* ES5 on methane production by hydrogenotrophic methanogens. Error bars represent standard deviations between duplicates.
